# Exploring Beekeepers’ Experiences and Perceptions of Anaphylaxis Risks: A Qualitative Study to Inform Targeted Health Education Programs

**DOI:** 10.3390/healthcare12242569

**Published:** 2024-12-20

**Authors:** Tea Močnik, Sabina Ličen, Mihaela Zidarn, Mirko Prosen

**Affiliations:** 1 Allergy Unit, University Clinic of Respiratory and Allergic Diseases Golnik, 4204 Golnik, Slovenia; mihaela.zidarn@klinika-golnik.si; 2 Faculty of Health Sciences, University of Primorska, 6310 Izola, Slovenia; sabina.licen@fvz.upr.si (S.L.); mirko.prosen@fvz.upr.si (M.P.); 3 Faculty of Medicine, University of Ljubljana, 1000 Ljubljana, Slovenia

**Keywords:** beekeepers, hypersensitivity reaction, epinephrine, exposure, awareness, prevention, health education

## Abstract

Background: Beekeeping plays crucial natural and economic roles but also poses health risks, as bee stings can cause severe allergic reactions like anaphylaxis, a potentially life-threatening condition that requires timely intervention. Understanding symptoms and the proper use of adrenaline autoinjectors is essential to minimize risks. This study aimed to assess the need for education on anaphylaxis and to develop a health education program to enhance beekeepers’ preparedness and safety. Methods: A qualitative descriptive interpretative method was employed. Two focus groups were conducted, one with eight health care professionals specializing in allergy and clinical immunology and the other with six active beekeepers. The data were analyzed via content analysis using QDA Miner^®^ Lite v3.0.5 software. Results: The analysis structure comprises five thematic areas: (1) the management of anaphylaxis; (2) the prevention of anaphylaxis; (3) health education approaches; (4) systemic approaches in prevention; and (5) adrenaline autoinjectors. The results highlight key challenges, including the need for better strategies to manage anaphylaxis, improve prevention, and provide practical educational programs for beekeepers. There is also a need for better collaboration between health care professionals and beekeepers, as well as improved access to and knowledge of adrenaline autoinjectors. Conclusions: Targeted education for beekeepers on recognizing anaphylaxis symptoms and using adrenaline autoinjectors is essential for timely intervention and preventing severe outcomes. Given their exposure to bee stings, beekeepers require proper training and regular practice to improve preparedness and safety. This research underscores the need for a comprehensive educational program to reduce anaphylaxis risk and enhance safety in beekeeping.

## 1. Introduction

Anaphylaxis is a severe systemic, potentially life-threatening hypersensitivity reaction. Approximately one-quarter of systemic reactions caused by Hymenoptera venom are severe. In the most serious cases, loss of consciousness can occur within minutes of the sting [1]. From 2010 to 2019, the Institute of Forensic Medicine in Slovenia conducted autopsies on 15 cases of death due to anaphylaxis. Among these deaths, 47% were the result of Hymenoptera stings. In all but one individual, the fatal case was recorded at the first instance of anaphylaxis [2], indicating the necessity of prevention, early recognition of clinical signs, and immediate intervention.

In the general population, bee stings are rare and usually occur only once every few years, depending on the individual’s occupation. Beekeepers often receive multiple bee stings each season and are among the at-risk populations for developing sensitization and, consequently, anaphylaxis [3,4]. Hypersensitivity to bee venom is frequently encountered in Slovenia, owing to the country’s long-standing beekeeping culture and large number of beekeepers. The beekeeping industry is comparable to other agricultural activities, highlighting the high awareness of the importance of bees and beekeeping [5,6].

Epinephrine autoinjectors are potentially life-saving and are intended for the immediate treatment of anaphylaxis [7,8,9]. They are designed to be easily used by laypeople. Notably, good education and the reinforcement of user knowledge are crucial for quick action [10,11,12,13]. In beekeeping, bee stings are possible and cannot be completely avoided even with the use of protective equipment. In many rural areas of Slovenia, quick access to emergency medical services is not always available. Given the increased risk of anaphylaxis and fatal outcomes for beekeepers, it would be sensible to equip beekeepers with epinephrine autoinjectors [14]. For the effectiveness of such a preventive approach, it is necessary to introduce education targeted to beekeepers to recognize the clinical signs of anaphylaxis and the correct use of epinephrine autoinjectors.

The existing data indicate the necessity of improving preventive measures and educational programs for individuals at risk of anaphylaxis. This research focuses on the development and implementation of effective strategies and health education programs aimed at reducing the incidence of anaphylaxis and increasing the safety of specific groups, such as beekeepers.

**Figure 1:**
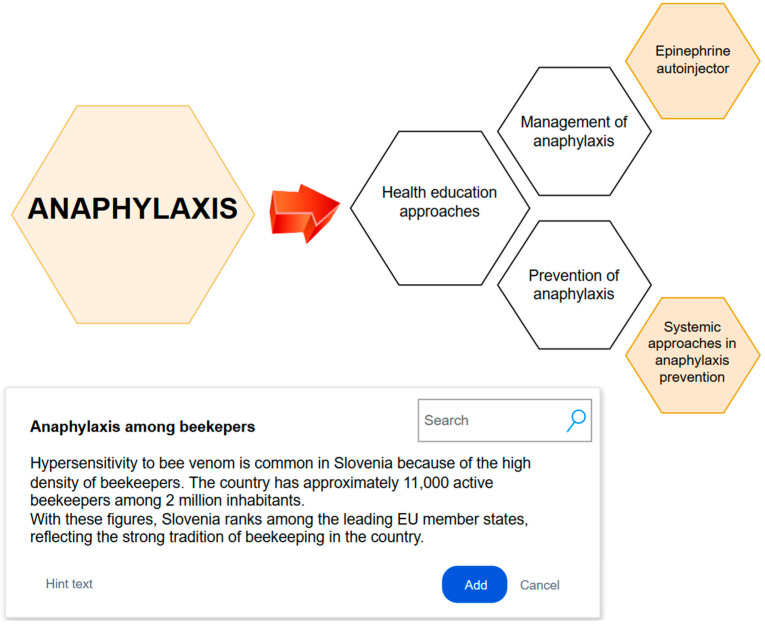
Diagram of key themes in anaphylaxis prevention and management among beekeepers: findings from focus group analysis.

The aim of this qualitative study was to explore the perspectives and experiences of beekeepers regarding anaphylaxis and the use of adrenaline autoinjectors. This study seeks to identify the need for educational interventions and to inform the development of an effective health education program aimed at improving the preparedness and safety of beekeepers. The key themes identified in the prevention and management of anaphylaxis among beekeepers, based on the focus group analysis, are presented in Figure 1.

Accordingly, the following research questions were formulated to guide the exploration: How do beekeepers perceive the risk of anaphylaxis? What are their experiences and challenges related to understanding prevention and the use of adrenaline autoinjectors? What are the viewpoints, professional guidelines, and experiences of experts regarding the prevention and management of anaphylaxis? Finally, how can the awareness and skills of beekeepers in managing anaphylaxis be enhanced, and what elements are essential in developing an effective health education program tailored to their needs?

## 2. Materials and Methods

### 2.1. Study Design

We used the descriptive-interpretative method [15], which allows for a detailed understanding and insight into the experiences and attitudes of health care professionals and is suitable for analyzing opinions on the implementation of a health education program focused on awareness, training, and the use of epinephrine autoinjectors in cases of anaphylaxis. This method is valuable for capturing the complexity of participants’ perspectives and provides a framework for interpreting phenomena within their contextual realities. An interpretive description is particularly effective in addressing the unique nature of issues in applied sciences, such as health care, given its ability to promote problem solving rather than merely generating new descriptions or theories. This approach aligns with the goals of our study, which aims to offer actionable insights for enhancing health education programs.

Qualitative research often requires close collaboration between the researcher and participants, as this allows for a deeper understanding of the experiences and perspectives of those involved in the research process. This collaboration typically develops through interaction and trust, enabling the researcher to gain valuable insights and information that would otherwise be difficult to obtain. By actively engaging with participants, researchers can adapt research methods and questions, leading to more relevant and meaningful findings [16].

### 2.2. Participants and Setting

Slovenian beekeeping has a long-standing tradition, emphasizing its significance for local agriculture and the environment. Today, approximately 11,000 beekeepers operate in Slovenia [6], highlighting the sector’s impact on preserving biodiversity and supporting sustainable agricultural practices. Following the recommendations of the authors, two large focus groups are sufficient for an in-depth understanding of the studied phenomenon, as additional focus groups do not significantly contribute to this understanding [17].

The expert group included 8 health care professionals with specialized knowledge in allergology and clinical immunology. The participants were purposively selected on the basis of their professional orientation in tertiary health care. Three participants were also actively involved in pedagogical activities. The participants were aged between 39 and 62 years and included 2 males and 6 females with various educational backgrounds, including 4 medical doctors and 4 registered nurses.

The group of beekeepers included 6 active beekeepers and members of the Slovenian Beekeepers’ Association. The participants ranged in age from 33 to 70 years, included 2 males and 4 females, and had various professional backgrounds (biologists, registered nurses, mechanical engineers, industrial designers and horticultural engineers, graduate heritage specialists, and doctors of pharmaceutical sciences).

### 2.3. Data Collection

Two measurement instruments were designed on the basis of a thematic guide for the focus group. The measurement instrument for experts includes 7 open-ended questions addressing the professional background of anaphylaxis, health care professionals’ experiences, and the evaluation of existing professional guidelines and challenges in the field of prevention. The instrument encourages the in-depth analysis of participants’ opinions and experiences, contributing to the understanding of key aspects of anaphylaxis prevention and the development of educational programs. Sample questions are presented in Table 1.

The measurement instrument for beekeepers contains 7 questions addressing the participants’ experiences in beekeeping, their attitudes towards and awareness of anaphylaxis, and their opinions on preventive health education programs regarding anaphylaxis. The instrument encourages the in-depth analysis of participants’ opinions and experiences, contributing to the understanding of key aspects of anaphylaxis prevention and the development of educational programs. Examples of these questions are presented in Table 2. The main questions are supplemented with subquestions to guide the participants.

### 2.4. Data Analysis

Audio recordings of the focus groups were transcribed verbatim. For qualitative data analysis, we used a combined approach that included initial coding and thematic analysis of each group separately to identify initial themes and patterns. After completing the separate analyses, we merged the codes of both groups. The content was analyzed using the QDA Miner^®^ software Lite v3.0.5. A cross-analysis technique was used to identify patterns and gain a deeper understanding. The participants’ names were replaced with specific codes (RN 1-4, MD 1-4, B 1-6). An interpretive approach was employed, allowing for a comprehensive understanding of the participants’ statements. Interpretive description represents an appropriate and theoretically flexible qualitative methodology that enables researchers to address complex experiential questions effectively and generate practical outcomes while maintaining methodological integrity [18]. The results were contextualized within the existing professional literature and identified important concepts for the development of preventive educational programs for beekeepers. Adherence to thematic analysis steps enabled a systematic approach to understanding the participants’ opinions in the focus groups, emphasizing the context of anaphylaxis prevention and the implementation of a health education program [16].

### 2.5. Rigor and Trustworthiness

To ensure the consistency and reliability of the results of this qualitative study, several key strategies were employed. The data were carefully documented and triangulated using various methods, such as focus groups and observations. This approach not only enhances the depth of the data but also provides a comprehensive view of the topic under investigation. The inclusion of different perspectives during the analysis allowed for a more nuanced understanding of the data and ensured that various viewpoints were considered. All stages of the research process were extensively documented, enabling traceability from data collection to final findings. Maintaining a reflective approach throughout the research process helped researchers remain aware of their own biases and assumptions that could influence data interpretation. By actively contemplating these influences, the researchers aimed to preserve the integrity of the study and produce findings that genuinely represent the experiences and perspectives of the participants.

### 2.6. Ethical Considerations

The ethics application for the study was approved by the Republic of Slovenia National Medical Ethics Committee (code: 0120-40/2023/6). The study was also approved by the members of the Research Council at the University Clinic of Respiratory and Allergic Diseases Golnik. All participants who agreed to participate provided their informed consent within the survey, acknowledging that anonymity within the focus group could not be guaranteed. However, all the data were anonymized before the analysis began.

## 3. Results

**Figure 2:**
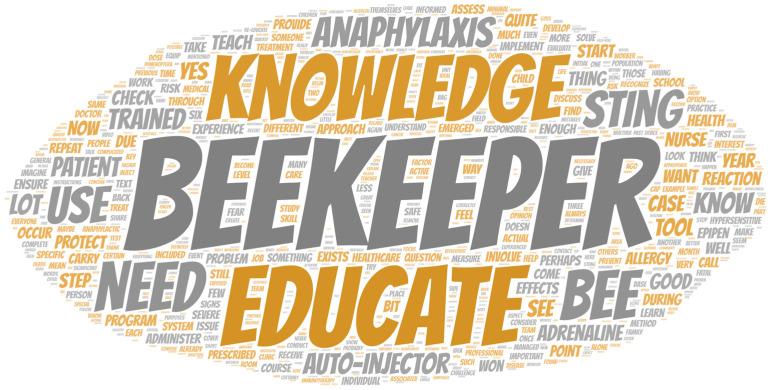
Word cloud of the most frequently used words in the focus groups.

The analysis yielded five thematic groups that highlight key aspects of anaphylaxis, risk recognition and management, and specific challenges that health care professionals believe are faced in the field of prevention: (1) the management of anaphylaxis; (2) the prevention of anaphylaxis; (3) health education approaches; (4) systemic approaches in prevention; and (5) the use of epinephrine autoinjectors (Table 3). The emphasis on education and awareness is crucial for reducing risk and effectively managing anaphylaxis, especially in high-risk groups such as beekeepers, as further illustrated in Figure 2, which presents the most frequently used words in the focus groups in a word cloud.

### 3.1. Management of Anaphylaxis

In the context of beekeeping, quick and effective responses to anaphylaxis are crucial for protecting the health of beekeepers. The participants initially emphasized the importance of quick and effective responses to anaphylaxis, including the administration of adrenaline. A medical doctor stated, “… equip the patient with an epinephrine autoinjector”. Another added, “For those who want to work more safely, offer quality training and this medication, which currently exists and they could have…” Anaphylaxis caused by bee stings poses a serious risk to the health of beekeepers. Adrenaline is the drug of choice for acute anaphylaxis and can quickly save lives. The participants in the study emphasized that the timely administration of adrenaline is essential for preventing severe consequences. When anaphylaxis occurs, swift action is the key; adrenaline autoinjectors must be kept on hand and used correctly. This allows beekeepers, who frequently face exposure to bee stings, to respond effectively and reduce the risk of fatal outcomes.

The discussion also addressed exposures and various risks associated with anaphylaxis and beekeeping. The participants highlighted the importance of awareness about specific risks to prevent anaphylaxis. A medical doctor noted, “They are a high-risk group”. Beekeepers are more frequently exposed to stings from Hymenoptera owing to the nature of their work, increasing the risk of developing hypersensitivity reactions. Stings from Hymenoptera can cause severe, even life-threatening anaphylaxis, especially when multiple stings occur simultaneously, resulting in larger doses of allergen. A medical doctor noted, “Stings from Hymenoptera are actually a leading cause of fatal anaphylaxis”, and another medical doctor added: “Beekeepers are more frequently exposed to these stings, and the greater the likelihood of stings, the greater the probability that a hypersensitivity reaction or allergy will develop”. With this awareness and education about the risks associated with anaphylaxis, beekeepers can better protect themselves and their health, contributing to greater safety in this profession.

Some participants noted the attitudes and awareness of beekeepers. Specifically, a medical doctor commented, “… with those I spoke to, they have already seen someone having a reaction, so I think everyone is a bit concerned”. A beekeeper agreed, “I think this is a fear for every beekeeper” (others nod in agreement). This dialog indicates that there is a certain awareness of the risks among beekeepers, but this awareness may not be sufficient to change behavior. Despite understanding potential dangers, many beekeepers express a strong desire to continue in their profession. As a medical doctor remarked, “They are a very special group of people, and most of them do not want to give up beekeeping”. This highlights the urgent need for effective educational programs that encourage the use of protective equipment and the adoption of preventive measures.

Increasing awareness of the severity of anaphylaxis, coupled with access to comprehensive educational resources, can significantly contribute to reducing the risk of anaphylaxis among beekeepers. Specific immunotherapy offers a long-term solution for managing hypersensitivity, but its effectiveness is closely linked to the avoidance of allergens. A medical doctor emphasized this point, stating, “In beekeepers who experience frequent stings even during immunotherapy, the treatment can become complicated, complications are more frequent, and the overall success of immunotherapy is diminished”. Thus, it is crucial to not only provide education on anaphylaxis and its management but also support beekeepers in implementing strategies that minimize their exposure to allergens, ensuring their health and safety in their role as stewards of the environment.

### 3.2. Prevention of Anaphylaxis

Effective preventive measures must be comprehensive and tailored to specific needs to ensure maximum efficacy. The participants further emphasized allergen avoidance as a key preventive strategy. A medical doctor noted, “In managing a patient with anaphylaxis or an allergic disease, the first priority is always ‘do everything to minimize allergen exposure’”. Another medical doctor added, “The first thing we always recommend is, of course, to stop beekeeping”. This confirms that allergen avoidance is the first step in managing patients with anaphylaxis. Preventing contact with allergens, such as stings from Hymenoptera, can significantly reduce the risk of anaphylaxis. Beekeepers should be aware that the environment in which they work poses inherent risks and that avoiding exposure to potential allergens is critical for their safety. This can be achieved through education on recognizing allergy symptoms and understanding the importance of avoiding situations that could lead to stings.

The discussion then addressed various challenges in implementing preventive measures. These challenges reflect the complexity of applying measures in practice, including logistical issues, lack of resources, or insufficient awareness. Medical doctors emphasized the following: “How to approach these individuals who are not yet patients but are likely to experience complications at some point?” and “Even children are beekeeping, beekeeping clubs are popular, so I think it would be quite important for all beekeepers to know that anaphylaxis exists, to recognize it, and to know how to respond properly”. Identifying these challenges is crucial for developing effective solutions. Successful preventive measures must be adapted to the specific needs of individuals and high-risk groups.

The importance of knowledge regarding the use of protective equipment among beekeepers was emphasized during the discussion. Although this topic was mentioned less frequently, it remains crucial for minimizing risks in specific occupational settings. A medical doctor noted, “Let’s start with personal protective equipment—it needs to be used correctly”. This sentiment was echoed by beekeepers themselves, with one stating, “When I work with bees, I’m one of those beekeepers who is always protected”. It is essential for beekeepers to receive education on the proper and consistent use of protective equipment, as this practice can significantly reduce the risk of anaphylaxis. By fostering a culture of safety and equipping beekeepers with the necessary knowledge, the likelihood of adverse health outcomes related to bee stings can be minimized, ultimately contributing to safer beekeeping practices.

### 3.3. Health Education Approaches

Emphasis on education, awareness, and support is crucial for reducing risk and improving the management of anaphylaxis. The participants focused on methods of health education for raising awareness about anaphylaxis. They highlighted the importance of informing patients about risks, symptoms, and emergency measures, including the use of autoinjectors for adrenaline. Registered nurses noted the following: “For patients identified with hypersensitivity, we provide education on allergen avoidance and the use of the EpiPen (n. adrenaline autoinjector)” and “We are the first contact, the ones who actually teach them. Therefore, health education is involved here”. Effective health education not only empowers patients but also strengthens their support systems. Groups at risk for anaphylaxis must be thoroughly educated on recognizing symptoms and taking appropriate action, including training their family members and colleagues, who may need to provide first aid in emergencies.

Various educational methods and adaptable techniques are important for reaching different goals or target groups. A registered nurse commented, “…not just brochures, just text, but there are manual skills, the perception, involving relatives”. Similarly, another registered nurse stated, “…I actually prefer training workshops”. The participants frequently emphasized the importance of practical exercises and involving family members in educational processes. Different approaches allow for tailoring content and teaching methods to the needs of various target groups, enhancing the effectiveness of educational programs. The use of various tools, such as training devices, can help participants gain practical experience and increase their confidence in using devices. Practical training combined with theory enables participants to face situations where they need to apply their knowledge and skills with greater confidence.

The need for precise knowledge assessment after participation in educational programs was highlighted. The high frequency of mentions confirms the need for systematic evaluation of knowledge. A registered nurse noted, “…we monitor this and appropriately score it, evaluate it in the information system”. This ensures that participants are trained and prepared to act in the event of anaphylaxis. Participants must be able to correctly identify the signs of anaphylaxis, know when to act, and know how to properly use the autoinjector for adrenaline. A participant added, “There are various tools that can be adjusted and used to control these manual skills in patients. There are questions about symptoms, when to act, timing, and whom to call”. The systematic evaluation of knowledge also allows for identifying areas needing further improvement.

Fear and lack of knowledge can seriously impact the effectiveness of preventive measures. A medical doctor noted the following: “If patients receive different instructions from different health care providers, patients become even more confused”. Another medical doctor added, “With our patients, we see that after a year, if not sooner, knowledge is no longer retained and what you did a year ago doesn’t count much anymore”. Fear of using the autoinjector for adrenaline, combined with a lack of knowledge, can seriously undermine the effectiveness of anaphylaxis management measures. To address these challenges, it is essential to develop strategies that not only educate patients but also build confidence in their use of life-saving devices.

Regular knowledge refreshment and practice are crucial for maintaining readiness and reducing the fear of using the autoinjector. Health care providers must ensure clear and consistent instructions to reduce confusion among patients. Registered nurses suggested the following: “Patients need re-education within two or three months” and “Certainly, it needs to be repeated continuously”. The importance of knowledge refreshment is also recognized by the beekeepers, one of whom stated, “And re-education, if you ask me, is needed at least once a year…/…because I’ve seen how important it is, you need to have that knowledge anchored”. Regular updates are particularly important for individuals at higher risk of anaphylaxis, as regular refreshment ensures that they are always prepared to act quickly and correctly.

The discussion of challenges and opportunities in education reflects the need for the ongoing adjustment and refinement of educational programs. A medical doctor commented, “The hardest part is teaching someone who hasn’t had a reaction yet”. Another medical doctor added, “It’s an opportunity for us to coordinate so that we all educate in the same way”. Challenges may include a lack of resources or appropriate methods, but opportunities offer possibilities for optimizing programs and improving the effectiveness of education. Successful education on anaphylaxis requires a comprehensive approach that includes coordination among various educational programs and provides consistent information to all participants. Opportunities for improving education may include developing new educational tools and methods tailored to the needs of different target groups.

### 3.4. Systemic Approaches for Anaphylaxis Prevention

The development and implementation of targeted preventive programs allows for a systematic approach to prevent anaphylaxis, which can increase the effectiveness of preventive measures and improve risk management. A registered nurse emphasized the following: “Our responsibility is to adequately empower them with knowledge”. This statement underscores the importance of education in preparing individuals to manage anaphylaxis. A medical doctor added, “Developing a simple yet highly effective system certainly plays an important role”. This confirms the need for straightforward but effective systems. A registered nurse suggested the following: “It’s a great opportunity for nurses in this field to empower these individuals”, highlighting that involving nurses in educational programs is crucial for improving prevention. Beekeepers also feel the need to develop and implement such programs. Specifically, a beekeeper noted, “You feel safer, and that’s the most important thing when you know how to react”.

The motivation to engage in preventive programs is the key to their success. Understanding the factors affecting participation is important for increasing the level of engagement and effectiveness of programs. A medical doctor noted, “Beekeepers have clearly expressed their desire for this”. Similarly, a beekeeper stated, “I believe that an enormous percentage of people will want to educate themselves about this”. This suggests that interest and willingness to learn among beekeepers are present, which is crucial for the successful implementation of programs.

The effectiveness of programs depends on the content and methods used. A medical doctor commented, “I think we need to start with what a normal response to a sting is, what a larger local reaction is, and then describe all the symptoms that can occur during anaphylaxis”. This indicates the importance of covering basic information about hypersensitivity reactions and anaphylaxis symptoms. Another medical doctor added the following: “First, they need to realistically understand the state or situation they are entering”. This means that participants must be aware of the risks beforehand. A registered nurse also mentioned the importance of “How to avoid allergens, how to use protective equipment, and how to properly use the EpiPen (autoinjector)”. A beekeeper emphasized the following: “I think it would be useful to also educate people on knowing the entire procedure, recognizing signs, and taking action”. These statements underscore the need for thorough training on allergen avoidance, the regular use of protective equipment, the recognition of systemic reaction signs, and the proper handling of autoinjectors.

Monitoring participants’ progress is important for the long-term success of programs. Although mentioned less frequently, ensuring that participants remain informed and trained is crucial. A doctor stated, “The way to maintain this knowledge should be through a lay system. That means through teams of the Beekeepers’ Association and, of course, with certification, so the Beekeepers’ Association can exclude those who would behave dangerously”. This indicates a need for an organized system for maintaining knowledge and tracking progress. Similarly, a beekeeper highlighted, “We already have a management system in place. We have a database of beekeepers who participate in our training”.

The discussion of obstacles and limitations in implementing preventive programs reflects the complexity of the challenges that may arise. Understanding these obstacles is crucial for finding solutions and improving processes. A medical doctor noted, “Unfortunately, it’s true that some solutions, known to be good in advance, are only implemented when a disaster occurs, not before”. This statement points to difficulties in implementing solutions before crisis situations arise. A systematic approach provides a clear overview of all important aspects of systemic approaches in anaphylaxis prevention and connects key quotes with interpretations for better understanding.

### 3.5. Epinephrine Autoinjector

Errors in the use of epinephrine autoinjectors have been identified as significant factors affecting the effectiveness of treatment. Medical doctors highlighted the following: “From this perspective, to encourage beekeepers to use it and to potentially give it to someone else. For example, if someone feels unwell, possibly owing to a vasovagal reaction, someone might decide to inject adrenaline”. and “There have been cases where patients primarily inject into their fingers”. These mistakes indicate the need for thorough training and ongoing education on the correct use of autoinjectors to reduce the risks of improper use and increase their effectiveness.

The participants also emphasized the importance of accessibility to autoinjectors for preventive purposes. A medical doctor stated, “They will have it if they are motivated, have completed the training, and know why they have it and when and how to use it”. The preventive role of autoinjectors is highlighted by all participants in the focus group of beekeepers, indicating that it is crucial for these devices to be accessible and for users to be well informed about their purpose and correct use. Increased awareness and accessibility can contribute to greater preparedness and a reduced risk of serious consequences.

Knowledge and confidence in the use of autoinjectors are recognized as keys to effective action in emergency situations. The participants emphasized that improving individuals’ confidence and readiness can significantly impact the success of managing anaphylaxis. A medical doctor commented, “We generally teach patients not to fear the administration of adrenaline”. This statement underscores the importance of ensuring that individuals feel confident in using autoinjectors, which is crucial for prompt and effective intervention in cases of anaphylaxis.

## 4. Discussion

This study demonstrated that timely intervention in anaphylaxis is crucial for successfully managing this life-threatening situation. Beekeepers, who are at greater risk of developing anaphylaxis due to their occupational exposure to bee stings, require adequate training to quickly recognize symptoms and take prompt action. For beekeepers who frequently encounter multiple stings each season, the risk of sensitization and subsequent development of anaphylaxis increases [4,19]. It is essential that beekeepers be well informed about preventive measures and the correct response in the event of an anaphylaxis. In Müller’s (2023) study, key risk factors for the development of allergic reactions to bee stings among beekeepers were identified, with the most important being the first years of beekeeping, the first stings in the spring, fewer than 10 bee stings per year, individuals with atopy, and those who show respiratory symptoms while working in the hive [20]. Also in the past, it was found that beekeepers often experience more severe reactions to bee stings, particularly in the spring and summer, and the importance of preventive measures during that time was highlighted [21]. Demirakle et al. (2018) examined the awareness of beekeepers and their family members about anaphylaxis and found that 55.1% of beekeepers are familiar with the term “anaphylaxis,” while 30.4% are aware of the epinephrine autoinjector. It is important that beekeepers, despite having basic knowledge of allergic reactions, are trained to recognize these symptoms and use the autoinjector, which can reduce the risk of severe consequences of anaphylaxis. The study emphasizes the need for education for beekeepers, particularly in terms of avoiding stings and taking immediate action in the event of anaphylaxis [22]. The mentioned studies emphasize the importance of raising awareness among beekeepers, and our work focuses on specific preventive strategies, including regular knowledge refreshers and practical exercises for managing anaphylaxis, which reduce the risk of fatal consequences from bee stings. Our research complements existing studies by identifying core themes for the development of a tailored educational program for beekeepers, based on the analysis of focus groups. In this way, we have contributed to the development of specific preventive strategies based on the needs of beekeepers, including regular knowledge updates, practical exercises for managing anaphylaxis, and the use of the epinephrine autoinjector when necessary.

Epinephrine, as the drug of choice, must be administered quickly and correctly to prevent serious consequences. Historically, research on the use of epinephrine autoinjectors has focused mainly on patients with previous anaphylaxis experience, but guidelines are now shifting towards ensuring access to epinephrine autoinjectors for potentially at-risk groups too. This means that beekeepers should also have access to autoinjectors and be able to use them quickly and easily in emergencies [8,14]. Importantly, proper education and the reinforcement of knowledge for users are crucial for the correct use of autoinjectors. Studies have shown that only 44% of patients with autoinjectors know how to use the device correctly. Sirin Kose et al. [12] reported that regular knowledge refreshment, at least every 12 months, is critical for recognizing clinical signs of anaphylaxis and for the timely and correct use of the epinephrine autoinjector. This is also supported by the opinions of the participants in our study. This highlights the need for regular education and training for beekeepers to increase their preparedness and ability to effectively manage emergencies. An appropriate and tailored educational strategy that incorporates simple and understandable methods can significantly reduce risk and improve the safety of beekeepers. Despite the existing awareness of risks, some beekeepers who are hypersensitive to venom do not choose to leave the profession, indicating a need for additional education and awareness. Avoiding allergens is a fundamental step in managing risk, but logistical challenges and a lack of resources often arise in implementing these measures. Educating beekeepers about the symptoms of anaphylaxis and emergency responses can significantly increase their readiness and reduce the risk of serious health complications. The inclusion of protective equipment usage in preventive strategies remains crucial for reducing risk and offering protection in specific work environments.

Systemic approaches to anaphylaxis prevention are essential for effectively managing risks. The development and implementation of targeted preventive health education programs that include training and education can greatly increase the effectiveness of preventive measures. McKenzie, Neiger, and Thackeray [23], in their book *Planning, Implementing, and Evaluating Health Promotion Programs*, emphasize that the successful implementation of preventive programs depends on accurately identifying the needs of the target group and effectively managing resources. In practice, simple and accessible strategies need to be developed for educating beekeepers that involve the use of educational methods that can be adapted to the specific needs of the group and any changes in the environment. The incorporation of practical exercises and regular knowledge refreshment are key to effective emergency preparedness. The authors also noted that successful program implementation requires coordination among different preventive program providers and the precise tracking of progress or knowledge evaluation. An effective training and progress monitoring system is crucial for the long-term success of preventive programs. The role of nurses in educational programs should also be highlighted, as their professional knowledge and experience can significantly contribute to the development of effective preventive strategies.

Research has confirmed that education, awareness, and a systematic approach are crucial for effectively managing anaphylaxis, especially among at-risk groups. The development and implementation of tailored educational programs that include regular knowledge updates, practical exercises, and appropriate access to autoinjectors are essential for reducing risk and increasing safety in beekeeping. A comprehensive and accessible approach to education and prevention can significantly contribute to improving preparedness and safety among specific high-risk groups.

Despite the significant insights gained from this research, certain limitations must be acknowledged. The study was based on a qualitative method using two focus groups, applying an interpretive description approach to enable an in-depth understanding of the topics [24]. However, the methodology may limit the breadth of findings to specific contexts and does not permit the quantitative validation of the results. The results are based on the subjective opinions and experiences of the participants, which may lead to interpretative differences compared with a broader population sample. Additionally, the research focuses on current guidelines for managing anaphylaxis and practices involving the use of adrenaline autoinjectors. Thus, the results may not be equally applicable to future changes in treatment guidelines. Although this research provides important guidelines for educating beekeepers about anaphylaxis and the use of adrenaline autoinjectors, the implementation and effectiveness of the recommended educational program have yet to be verified. The educational program developed on the basis of the results will require testing and evaluation before it can be established as an effective approach at the national level. Experts in the focus groups are unanimous in their opinion that anaphylaxis must be treated with the immediate administration of epinephrine. However, they emphasize that merely increasing access to epinephrine autoinjectors is not sufficient to improve the safety of beekeepers, who are at higher risk of anaphylaxis. The focus is placed on developing a targeted educational program, where participants would be trained not only to recognize the signs of anaphylaxis but also to take immediate action, including the correct use of the adrenaline autoinjector. In this way, the aim is to ensure that beekeepers are not only prepared with access to the necessary treatment but also possess the knowledge and skills to act effectively in emergencies, thereby improving their overall safety.

## 5. Conclusions

The prevention of anaphylaxis in beekeepers involves targeted education, awareness, and a systematic approach. Owing to their occupational exposure to bee stings, beekeepers are at increased risk of developing anaphylaxis. Therefore, it is crucial for beekeepers to be adequately trained to quickly recognize and respond to potential reactions. The implementation of a comprehensive health education program, which includes tailored educational content, regular knowledge refreshment, and effectiveness evaluation, is essential for enhancing the safety of beekeepers and reducing the risk of anaphylaxis.

## Figures and Tables

**Table 1:** Sample questions for health care professionals on anaphylaxis prevention and education.

Question	Topic
What is your professional background or opinion on anaphylaxis and the treatment of systemic hypersensitivity reactions specifically related to beekeeping?	Professional background and opinions on anaphylaxis in the context of beekeeping.
How are health education approaches conducted for patients dealing with hypersensitivity to bee venom?	Current health education approaches for hypersensitivity to bee venom.
What is your opinion on the existing health education approaches used for the prevention of anaphylaxis in at-risk groups?	Evaluation of existing approaches for anaphylaxis prevention in at-risk groups.
What do you think about implementing a new educational program tailored to the specific needs of beekeepers, including recognizing signs of anaphylaxis and appropriate actions?	Implementation of a new program for beekeepers focusing on recognizing and managing anaphylaxis.
Which topics do you believe should be included in such training from a health education perspective?	Suggested topics for inclusion in health education training.
How would you assess the acquired knowledge and manual skills after completing the health education program?	Assessment of knowledge and skills post-training.
What do you consider to be the most critical reasons for integrating a preventive program into the existing educational structure?	Importance of integrating a preventive program into existing educational structures.

**Table 2:** Sample questions for beekeepers on their experiences and perceptions of anaphylaxis and prevention.

Question	Topic
How does your work typically proceed in the beekeeping environment?	Work procedures and environment in beekeeping.
What actions do you take in response to a bee sting or multiple stings during your work?	Immediate actions taken in response to bee stings.
Do you believe it is possible to develop hypersensitivity to bee venom over time, and how do you perceive this risk?	Perceptions of risk related to developing hypersensitivity to bee venom.
How would you handle a situation where you or someone close to you experienced a systemic allergic reaction?	Response to a systemic allergic reaction in oneself or close individuals.
What is your opinion on implementing a health education program aimed at recognizing systemic hypersensitivity reactions to prevent anaphylaxis?	Opinion on implementing a health education program for anaphylaxis prevention.
What topics do you think should be included in such a training program?	Suggested topics for inclusion in a health education training program.
How do you view the availability of adrenaline autoinjectors for preventive use among at-risk groups?	Perceptions of the availability and use of adrenaline autoinjectors for at-risk groups.

**Table 3:** Themes, subthemes, and codes.

Theme	Subtheme	Code
Management of anaphylaxis	Recognition and response to anaphylaxis	MNG-01
	Exposure and risk of anaphylaxis	MNG-02
	Beekeeper experiences, awareness, and attitudes	MNG-03
	Treatment with specific immunotherapy	MNG-04
Prevention of anaphylaxis	Avoidance of allergen	PREV-01
	Challenges in anaphylaxis prevention	PREV-02
	Personal protective equipment for beekeeping	PREV-03
Health education approaches	Implementation of health education	EDU-01
	Methods and techniques of education	EDU-02
	Evaluation and methods of knowledge assessment	EDU-03
	Lack of knowledge, fear, and poor adherence	EDU-04
	Knowledge refreshment	EDU-05
	Challenges and opportunities in education	EDU-06
Systemic approaches in anaphylaxis prevention	Needs, benefits, and the importance of developing and implementing health education programs	SYS-01
	Motivation for implementing preventive health education programs	SYS-02
	Topics and methods of implementing preventive health education programs	SYS-03
	Traceability	SYS-04
	Barriers and limitations	SYS-05
Epinephrine autoinjector	Epinephrine autoinjector equipment	EPI-01
	Errors in handling epinephrine autoinjectors	EPI-02
	Availability and accessibility of epinephrine autoinjectors for prevention	EPI-03
	Knowledge, confidence, and preparedness to act	EPI-04

## Data Availability

The data supporting the findings of this study are available from the corresponding author upon reasonable request.

## References

[1] Turner P.J., Jerschow E., Umasunthar T., Lin R., Campbell D.E., Boyle R.J. (2017). Fatal Anaphylaxis: Mortality Rate and Risk Factors. J. Allergy Clin. Immunol. Pract..

[2] Rijavec M., Inkret J., Bidovec-Stojković U., Carli T., Frelih N., Kukec A., Korošec P., Košnik M. (2023). Fatal Hymenoptera Venom–Triggered Anaphylaxis in Patients with Unrecognized Clonal Mast Cell Disorder—Is Mastocytosis to Blame?. Int. J. Mol. Sci..

[3] Stanhope J., Carver S., Weinstein P. (2017). Health outcomes of beekeeping: A systematic review. J. Apic. Res..

[4] Worm M., Moneret Vautrin A., Scherer K., Lang R., Fernandez Rivas M., Cardona V., Kowalski M.L., Jutel M., Poziomkowska-Gesicka I., Papadopoulos N.G. (2014). First European data from the network of severe allergic reactions (NORA). Allergy.

[5] Chauzat M.P., Cauquil L., Roy L., Franco S., Hendrikx P., Ribière-Chabert M. (2013). Demographics of the European apicultural industry. PLoS ONE.

[6] Slovenian Government Slovenia and Beekeeping. https://www.gov.si/en/registries/projects/world-bee-day/slovenija-in-cebelarstvo/.

[7] Bilò M.B., Cichocka-Jarosz E., Pumphrey R., Oude-Elberink J.N., Lange J., Jakob T., Bonadonna P., Fernandez J., Kosnik M., Helbling A. (2016). Self-medication of anaphylactic reactions due to Hymenoptera stings-an EAACI Task Force Consensus Statement. Allergy.

[8] Muraro A., Worm M., Alviani C., Cardona V., DunnGalvin A., Garvey L.H., Riggioni C., de Silva D., Angier E., Arasi S. (2022). EAACI guidelines: Anaphylaxis (2021 update). Allergy.

[9] Song T.T., Worm M., Lieberman P. (2014). Anaphylaxis treatment: Current barriers to adrenaline auto-injector use. Allergy.

[10] Ediger D., Terzioglu K., Ozturk R.T. (2018). Venom allergy, risk factors for systemic reactions and the knowledge levels among Turkish beekeepers. Asia Pac. Allergy.

[11] Kessler C., Edwards E., Dissinger E., Sye S., Visich T., Grant E. (2019). Usability and preference of epinephrine auto-injectors: Auvi-Q and EpiPen Jr. Ann. Allergy Asthma Immunol..

[12] Sirin Kose S., Asilsoy S., Tezcan D., Al S., Atay O., Kangalli O., Uzuner N., Karaman O. (2020). Is there an optimal training interval to improve the correct use of adrenaline auto-injectors?. Int. Arch. Allergy Immunol..

[13] Kadivec S., Košnik M. (2021). The ability to use epinephrine autoinjector in patients who receive prescription immediately after anaphylaxis. Int. Arch. Allergy Immunol..

[14] Sato K., Hirata H., Tatewaki M., Shiromori S., Souma R., Satoh H., Sugiyama K., Arima M., Kurasawa K., Fukuda T. (2020). Emergency Treatment of Anaphylaxis in Japanese Beekeepers. J. Agromed..

[15] Elliott R., Timulak L. (2021). Essentials of Descriptive-Interpretive Qualitative Research: A Generic Approach.

[16] Peel K.L. (2020). A beginner’s guide to applied educational research using thematic analysis. Pract. Assess. Res. Eval..

[17] Hennink M.M., Kaiser B.N., Weber M.B. (2019). What influences saturation? Estimating sample sizes in focus group research. Qual. Health Res..

[18] Thompson Burdine J., Thorne S., Sandhu G. (2021). Interpretive description: A flexible qualitative methodology for medical education research. Med. Educ..

[19] Ruëff F., Bauer A., Becker S., Brehler R., Brockow K., Chaker A.M., Darsow U., Fischer J., Fuchs T., Gerstlauer M. (2023). Diagnosis and treatment of Hymenoptera venom allergy: S2k Guideline of the German Society of Allergology and Clinical Immunology (DGAKI) in collaboration with the Arbeitsgemeinschaft für Berufs- und Umweltdermatologie e.V. (ABD), the Medical Association of German Allergologists (AeDA), the German Society of Dermatology (DDG), the German Society of Oto-Rhino-Laryngology, Head and Neck Surgery (DGHNOKC), the German Society of Pediatrics and Adolescent Medicine (DGKJ), the Society for Pediatric Allergy and Environmental Medicine (GPA), German Respiratory Society (DGP), and the Austrian Society for Allergy and Immunology (ÖGAI). Allergol. Sel..

[20] Müller U.R. (2005). Bee venom allergy in beekeepers and their family members. Curr. Opin. Allergy Clin. Immunol..

[21] Leclercq M., Bosson L., Lecomte J. (1982). Les apiculteurs et les piqûres d’hyménoptères: Résultats d’une enquête. Rev. Médicale De Liège.

[22] Demirkale Z.H., Yücel E., Çimen S.S., Süleyman A., Özdemir C., Kara A., Tamay Z. (2020). Venom allergy and knowledge about anaphylaxis among beekeepers and their families. Allergol. Et Immunopathol..

[23] McKenzie J.F., Neiger B.L., Thackeray R. (2022). Planning, Implementing and Evaluating Health Promotion Programs.

[24] Iqbal S., Ladak L., Ali H., Barolia R. (2023). Interpretive Description Research: A Futile or Fertile Value Add to Nursing Science. Rehman J. Health Sci..

